# An Investigation of the Highly Stable Interface in Zn^2+^/Mn^2+^-EG-Based Deep Eutectic Electrolytes for Zinc-Ion Batteries

**DOI:** 10.3390/nano16060342

**Published:** 2026-03-10

**Authors:** Jiangjin Hou, Xinyu Yan, Xiling Mao, Kaihua Yao, Xiangyang Xin, Mengwei Li

**Affiliations:** School of Instrument and Electronics, North University of China, Taiyuan 030051, China; houjiangjin@163.com (J.H.); xlmao2014@163.com (X.M.); yaokaihua6@163.com (K.Y.); xinxiangyang95@163.com (X.X.)

**Keywords:** zinc-ion batteries, deep eutectic solvent, solvation shell reconstruction ethylene glycol, dendrite-free deposition

## Abstract

Zinc-ion batteries have garnered significant research interest owing to their inherent safety, low cost, and environmental compatibility. Nevertheless, their widespread adoption is impeded by critical challenges including uncontrollable dendrite growth, parasitic side reactions stemming from active water molecules, and the corrosion of the zinc anode in conventional aqueous electrolytes. Herein, a hydrated deep eutectic solvent (HDES) electrolyte based on ZnSO_4_, MnSO_4_, and ethylene is proposed for high-performance zinc-ion batteries. This electrolyte demonstrates excellent stability and simultaneously enables the formation of a protective coating on the Zn anode surface. Spectroscopic analyses and theoretical simulations reveal that this electrolyte reconfigures the primary Zn^2+^ solvation shell by replacing water molecules with HDES components. This tailored solvation structure facilitates interfacial desolvation, elevates nucleation overpotential, and promotes uniform, dendrite-free zinc deposition. Simultaneously, a robust hydrogen bond network effectively sequesters free water, significantly suppressing the hydrogen evolution reaction and anode corrosion. Benefiting from these features, the HDES-based full cell delivers exceptional long-term stability, achieving over 2000 cycles at 3 mA cm^−2^ with a capacity retention exceeding 95% and a Coulombic efficiency surpassing 85%. In sharp contrast, the traditional aqueous counterpart fails within only 200 cycles. This tenfold lifespan enhancement, coupled with cost-effectiveness and non-flammability, presents a promising strategy for advanced, grid-scale zinc-based energy storage.

## 1. Introduction

With the accelerating global transition towards carbon neutrality, the demand for reliable, cost-effective, and safe electrochemical energy storage systems has reached unprecedented levels [1,2,3,4]. ZIBs have emerged as a formidable candidate for next-generation energy storage systems. Their global appeal stems from a unique combination of merits, including cost-effectiveness, environmental sustainability, intrinsic safety, and high volumetric capacity (5855 mAh cm^−3^), all while maintaining compatibility with aqueous electrolytes [5,6,7]. As an indispensable constituent, the electrolyte exerts a profound influence on the overall electrochemical performance and longevity of ZIBs [8]. They ensure a stable electrochemical potential window (1.0–2.0 V), facilitate Zn^2+^ transport between the anode and cathode, and fundamentally dictate both the reaction mechanism and ionic conductivity [9].

Aqueous zinc-ion batteries (AZIBs) offer a range of advantages, most notably their high energy density (200–250 Wh kg^−1^) [10,11], intrinsic safety, excellent long-term cycling stability, and environmental benignity (>1000 cycles) [12,13,14]. However, the thermodynamic instability of water molecules within traditional dilute aqueous electrolytes constitutes a primary bottleneck. Under the electrochemical environment of ZIBs, free water molecules are prone to decomposition due to the narrow stability window of water (1.23 V) [15,16,17]. In neutral or mildly acidic media, this manifests as parasitic hydrogen evolution reactions (HERs) and electrode corrosion, leading to the continuous consumption of the electrolyte and the accumulation of insulating byproducts (e.g., basic zinc sulfates) [18]. More critically, the uneven flux of Zn^2+^ ions and the “tip effect” of the electric field on the anode surface inevitably induce the uncontrollable growth of zinc dendrites [19]. These sharp dendrites not only accelerate capacity decay by forming “dead zinc” but also pose a severe safety hazard by piercing the separator, causing catastrophic short circuits [20].

To circumvent these water-induced limitations, significant research efforts have been dedicated to modulating the Zn^2+^ solvation environment. “Water-in-salt” (WIS) electrolytes, which utilize super-concentrated salts (e.g., Zn(TFSI)_2_ or LiTFSI) to bind free water, have proven effective in suppressing parasitic reactions and widening the electrochemical window [21,22,23,24]. Similarly, Room-Temperature Ionic Liquids (RTILs), particularly imidazolium-based systems, demonstrate exceptional kinetics and stability [25]. However, the practical scalability of these advanced strategies is often constrained by prohibitive costs, high viscosity, severe moisture sensitivity, and the toxicity of fluorinated salts [26]. While various organic and inorganic additives have been employed to regulate nucleation [27,28], their synthesis is often intricate, and they typically fail to fundamentally resolve the thermodynamic activity of bulk water.

To address these limitations, DESs—a class of innovative ionic fluids analogous to ionic liquids—have rapidly emerged as promising electrolyte candidates [29,30,31]. Typically synthesized by complexing Lewis acids and bases at specific molar ratios to form eutectic mixtures via extensive hydrogen bonding networks, DESs are renowned for their low volatility, biodegradability, and unique interfacial properties. Based on their physicochemical properties, DESs can be broadly categorized into acidic, alkaline, and neutral types. Acidic DESs (e.g., formed with carboxylic acids) and alkaline DESs often exhibit high reactivity, which may lead to the severe corrosion of battery components. In contrast, neutral DESs, typically composed of metal salts and neutral hydrogen bond donors (e.g., amides or polyols), offer a mild electrochemical environment. Therefore, neutral DESs are increasingly recognized as environmentally benign “green” solvents that bridge the gap between performance and economic feasibility, making them ideal for stabilizing zinc anodes [32,33].

Herein, we report a cost-effective, eco-friendly, and intrinsically safe neutral zinc sulfate/manganese sulfate/ethylene glycol HDES (ZnSO_4_–MnSO_4_–EG) system as an electrolyte for high-performance ZIBs [34,35]. In this system, EG serves as a pivotal “water blocker” that effectively “locks” free water by establishing a strongly interacting hydrogen bond network. This mechanism creates a confined high-concentration electrolyte environment that facilitates a protective coating, effectively alleviating surface corrosion and the HER. Through a combination of theoretical simulations and experimental characterizations, we demonstrate that this electrolyte reconstructs the Zn^2+^ solvation sheath and optimizes its coordination environment [36]. This novel coordination structure not only minimizes disorder during Zn deposition but also refines the grain size of the deposition products by elevating the nucleation overpotential, thus suppressing the growth of stubborn dendrites at the source [2,37]. Benefiting from optimized interfacial protection and accelerated desolvation kinetics, ZIBs based on this HDES exhibit superior electrochemical performance, achieving a lifespan of over 2000 cycles (a 10-fold increase compared to traditional aqueous electrolytes), with capacity retention stabilized above 95%. This work not only demonstrates the high efficiency of this multifunctional electrolyte system but also offers new perspectives and possibilities for developing a family of low-cost, highly safe non-aqueous electrolytes suitable for grid-scale energy storage applications.

## 2. Materials and Methods

### 2.1. Preparation

Zinc sulfate heptahydrate (ZnSO_4_·7H_2_O, reagent grade, 99.5%), manganese sulfate monohydrate (MnSO_4_·H_2_O, reagent grade, 99%), and ethylene glycol (analytically pure, 99%) were purchased from Shanghai Macklin Biochemical Co., Ltd. (Shanghai, China). Anhydrous ethanol (analytically pure, 99.7%) was obtained from Sinopharm Chemical Reagent Co., Ltd. (Shanghai, China). Polyvinylidene fluoride (PVDF) was sourced from Shanghai Aladdin Biochemical Technology Co., Ltd. (Shanghai, China), and conductive carbon black (Super P Li) was supplied by Guangdong Canrd New Energy Technology Co., Ltd. (Dongguan, China).

**Figure 1:**
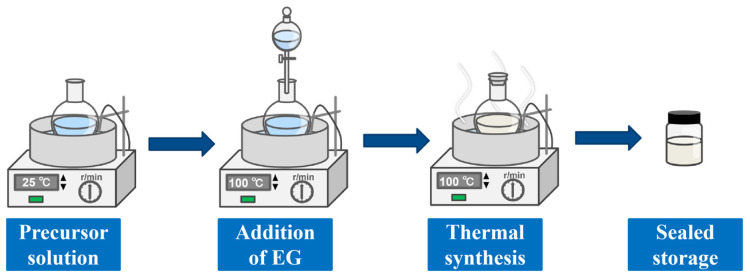
Schematic illustration of HDES preparation.

The HDES electrolytes were synthesized by incorporating metal salt hydrates into a robust hydrogen bonding network. The preparation process of the HDES is shown in Figure 1. As is typical, a concentrated precursor solution containing ZnSO_4_ and MnSO_4_ (molar ratio 10:1) was first prepared. EG was subsequently added dropwise into the mixture under continuous magnetic stirring in an oil bath to facilitate the formation of an extensive hydrogen bonding network among the metal cations (Zn^2+^/Mn^2+^), water molecules, and EG molecules. To optimize the electrolyte formulation, the molar ratios of ZnSO_4_ to EG were systematically adjusted (1:3, 1:4, and 1:5). Synthesis was conducted at various temperatures (80 °C, 100 °C, and 120 °C) with stirring durations of 30 min, 60 min, and 90 min under atmospheric pressure. Stirring was maintained until a transparent, homogeneous liquid was obtained, signaling the successful formation of the HDES. Finally, the synthesized electrolytes were sealed and stored in glass vials for further characterization.

The working electrodes for the three-electrode configuration were fabricated by mixing MnO_2_, conductive carbon black, and PVDF binder in a mass ratio of 8:1:1. The mixture was dispersed (typically in N-methyl-2-pyrrolidone) to form a homogeneous slurry, which was then uniformly coated onto a carbon cloth substrate ($1\times 1{\;\mathrm{cm}}^{2}$). The typical mass loading of the active material was maintained at approximately 1 mg. Subsequently, the prepared electrodes were dried in a vacuum oven at 70 °C for 12 h to ensure the complete removal of the solvent.

Fabrication of asymmetric Zn-MnO_2_ full cells: The MnO_2_ cathodes were fabricated by blending manganese dioxide, conductive carbon black, and PVDF binder in a mass ratio of 8:1:1. The mixture was dispersed (typically in N-methyl-2-pyrrolidone) to form a homogeneous slurry, which was then uniformly coated onto a titanium (Ti) foil substrate with an effective area of $3\times 3{\;\mathrm{cm}}^{2}$. The mass loading of the MnO_2_ active material was controlled between 10 and 15 mg. Subsequently, the prepared electrodes were dried in a vacuum oven at 70 °C for 12 h to ensure the complete removal of the solvent.

Battery assembly: A MnO_2_//Zn asymmetric cell was fabricated in an electrolytic cell, comprising a Zn foil negative electrode, a MnO_2_ positive electrode, and a HDES electrolyte. This setup was employed to elucidate the charge storage mechanism of the asymmetric device in the HDES system.

### 2.2. Characterization

The morphology of the samples was characterized using field emission scanning electron microscopy (FE-SEM). Fourier transform infrared (FT-IR) spectroscopy (Nicolet iS20, Thermo Scientific, Waltham, MA, USA) was employed to analyze the molecular structures and functional groups based on the positions and intensities of characteristic absorption peaks. The thermal stability of the synthesized HDES was investigated using a thermogravimetric analyzer (Themys, Setaram, France). Furthermore, the viscosity of the prepared liquid was measured using a rheometer (Anton Paar MCR 302, manufactured by Anton Paar GmbH, Graz, Austria).

### 2.3. Model Implementation

This mechano-electrochemical model was simulated by the finite element method on COMSOL Multiphysics 6.3. To simplify the model, a 5 μm × 6 μm electrolyte region was constructed for the simulation. The simulation considers two different samples: the first electrolyte is 2 mol/L ZnSO_4_, and the second electrolyte is a mixed solution of 2 mol/L ZnSO_4_, 0.2 mol/L MnSO_4_, and ethylene glycol in a molar ratio of 10:1:40. By simulating the growth morphology of dendrites in these two electrolytes, the differences between the two electrolytes are compared [38].

The simulation steps include a transient step. The surfaces of all negative electrodes are applied with 0 V electric potential, and the positive electrode surfaces are applied with 0.1 V electric potential. The initial Zn^2+^ ion concentration in the electrolyte is set to 1000 mol/m^3^. The Zn plating time is set as 300 s.

Model Formulation:

A typical cell, which consists of a Zn metal electrode and electrolyte (including solid electrolyte and electrolyte-immersed separator), was employed as the research system. Two phases and three components in this system are distinguished by a non-conserved order parameter ξ (electrolyte, ξ = 0; Zn metal anode, ξ = 1) and a concentration set c_i_ (i = Zn, Zn^2+^, and anion), respectively. The local electrostatic potential is denoted as ϕ_i_ (i = Zn and e, representing Zn metal electrode and electrolyte, respectively), and the displacement field is represented by u. The total free energy of this system is given by

(1) $$F=\int_{V}\left(f_{\mathrm{grad}}\left(\xi \right)+f_{\mathrm{ch}}\left(\xi ,c_{i}\right)+{\;f}_{\mathrm{elec}}\left(\xi ,c_{i},\phi_{i}\right)+f_{\mathrm{els}}\left(\xi ,u\right)\right)\mathrm{dV}$$

where f_grad_, f_ch_, f_elec_, and f_els_ represent the local energy density from the gradient, chemical, electrostatic and elastic contribution, respectively [39].

The electrochemical reaction (Zn^2+^ + e^2−^ → Zn) under the driving force of Equation (4) can be deduced from the Butler−Volmer equation. It is expressed by

(2) $$\frac{\partial \xi }{\partial t}=-L_{\sigma }\left(g^{\prime }\left(\xi \right)+f_{\mathrm{grad}}^{\prime }\left(\xi \right)+f_{\mathrm{els}}^{\prime }\left(u,\xi \right)\right)-L_{\eta }h^{\prime }\left(\xi \right)\left(e^{\frac{\left(1-\alpha \right)F\eta }{\mathrm{RT}}}-\frac{C_{{\mathrm{Zn}}^{2+}}}{C_{0}}e^{\frac{-\alpha F\eta \;}{\mathrm{RT}}}\right)$$

where L_σ_ is the interfacial mobility, L_η_ the reaction constant, α and 1 − α the charge transfer coefficient, and C_0_ the initial concentration of electrolyte. h (ξ) = ξ^3^ (6 × ξ^2^ − 15 × ξ + 10) is an interpolating function. η = φ_Zn_ − φ_e_ − E_eq_ is the overpotential, where φ_Zn_ denotes the potential of the Zn metal anode, φ_e_ the potential of the electrolyte, and E_eq_ the equilibrium potential of electrochemical reaction [40].

Therefore, the evolution of C_Zn2+_ in the electrolyte can be described by the Nernst−Planck equation,

(3) $$\frac{\partial C_{{\mathrm{Zn}}^{2+}}}{\partial t}=\nabla \cdot \left(D_{{\mathrm{Zn}}^{2+}}\nabla_{C_{{\mathrm{Zn}}^{2+}}}+D_{{\mathrm{Zn}}^{2+}}C_{{\mathrm{Zn}}^{2+}}\frac{F}{\mathrm{RT}}\nabla \phi_{e}\right)-C_{\mathrm{Zn}}\frac{\partial \xi }{\partial t}$$

where D_Zn2+_ represents the diffusion coefficient of Zn^2+^ and C_Zn_ the initial concentration of the electrode. Both diffusion and electromigration are considered. The electrostatic potential distribution can be expressed by

(4) $$\nabla \cdot \left(-\sigma_{\mathrm{eff}}\nabla \phi_{e}\right)=0$$

(5) $$\nabla \cdot \left(\sigma_{\mathrm{eff}}\nabla \phi_{\mathrm{Zn}}\right)=FC_{\mathrm{Zn}}\frac{\partial \xi }{\partial t}$$

Herein, σ_eff_ = h(ξ)σ_Zn_ + (1 − h(ξ))σ_e_ is the effective electric conductivity, where σ_Zn_ and σ_e_ represent the electric conductivity of the electrode and electrolyte, respectively.

### 2.4. Electrochemical Measurements

Electrochemical measurements were performed on a CHI660E electrochemical workstation (Shanghai, China) using a standard three-electrode configuration. MnO_2_ coated on carbon cloth served as the working electrode, while a zinc foil and a silver/silver chloride (Ag/AgCl) electrode were employed as the counter and reference electrodes, respectively. The synthesized HDES was used as the electrolyte. All tests were conducted at ambient temperature. The electrochemical properties of the HDES electrolytes, prepared under varying synthesis conditions—including varying temperatures (80 °C, 100 °C, and 120 °C), molar ratios (1:3, 1:4, and 1:5), and durations (30 min, 60 min, and 90 min)—were systematically evaluated and compared via cyclic voltammetry (CV), galvanostatic charge/discharge (GCD), and electrochemical impedance spectroscopy (EIS).

GCD measurements were performed using a Neware battery testing system. Cycling stability was evaluated over 2000 cycles within a potential window of 1.2–2.1 V at a current density of 3 mA cm^−2^. All electrochemical experiments were conducted at ambient temperature.

## 3. Results and Discussion

### 3.1. Theoretical Simulation and Morphological Analysis

To gain deeper insights into the zinc deposition kinetics across various electrolyte systems, an electrochemical deposition model was developed based on the phase-field method. In this model, a non-conserved order parameter ξ (ξ = 0 representing the electrolyte phase and ξ = 1 representing the zinc metal phase) was introduced to track the evolution of the electrode/electrolyte interface. The total free energy F of the system is collectively determined by the gradient, chemical, electrostatic, and elastic energies (Equation (1)). The transport behavior of zinc ions is governed by the Nernst–Planck equation (Equation (3)), which accounts for the interplay between diffusion (D_Zn2+_∇C) and electromigration (D_Zn2+_ C$\frac{F}{\mathrm{RT}}$∇ϕ). Meanwhile, the interfacial electrochemical reaction rate is described by Butler–Volmer kinetics (Equation (2)).

**Figure 2:**
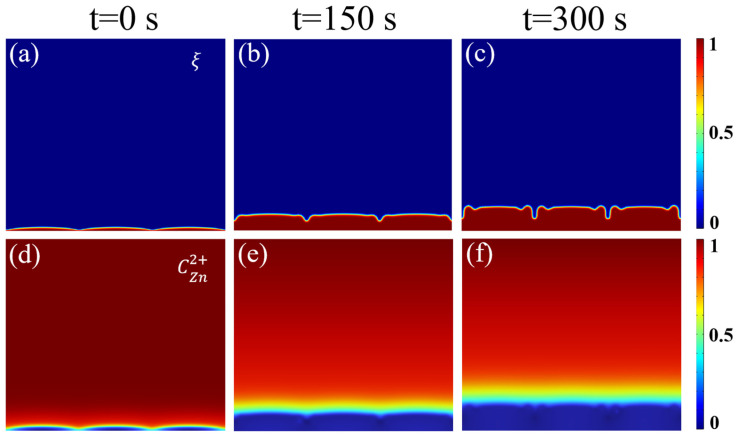
(**a**–**c**) The phase-field simulation results of dendrite growth on the Zn anode in the HDES system from 0 to 300 s; (**d**–**f**) corresponding Zn-ion concentration distributions.

Figure 2 illustrates the simulation and experimental results within the HDES electrolyte system. According to the interfacial evolution captured by phase-field simulations (Figure 2a–c), the zinc anode maintains a predominantly flat, layered growth mode throughout the deposition process from t = 0 to 300 s. Although slight curvature fluctuations occur on the surface, only rounded protrusions are formed even in the late stages of deposition, with no observable sharp dendritic growth.

The corresponding concentration field simulations (Figure 2d–f) reveal the underlying mechanism: in the HDES system, the concentration gradient lines remain highly parallel to the electrode surface, exhibiting no significant ionic “hotspots” or severe distortions even at the convex sites. This indicates that the HDES system possesses exceptional ion transport characteristics, providing a uniform ion flux that effectively suppresses the local ionic accumulation induced by the electromigration term in the Nernst–Planck equation, thereby eliminating the “tip effect.”

**Figure 3:**
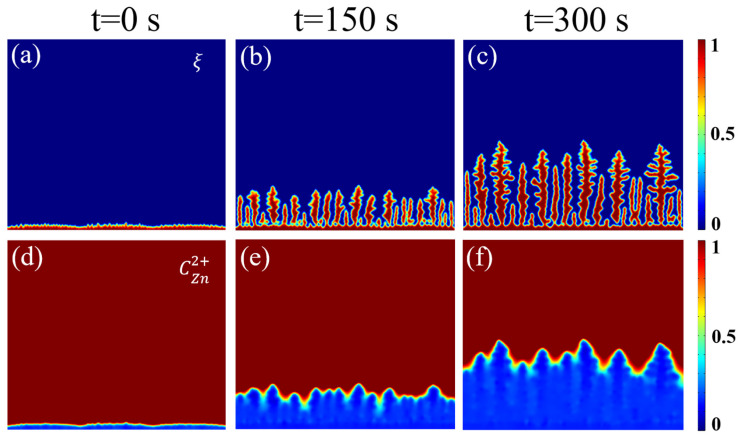
(**a**–**c**) The phase-field simulation results of Zn dendrite growth in the aqueous ZnSO_4_ system from 0 to 300 s; (**d**–**f**) Zn-ion concentration distributions.

Figure 3 illustrates the “runaway” deposition behavior observed in the conventional aqueous ZnSO_4_ electrolyte. Phase-field simulations (Figure 3a–c) reveal that sharp vertical protrusions emerge at the interface as early as t = 150 s, exhibiting a strong tendency for preferential growth. By t = 300 s, these regions have evolved into dense lamellar structures.

The corresponding concentration field simulations (Figure 3d–f) uncover the root cause of this failure: restricted ion diffusion in the aqueous electrolyte leads to the formation of an ion enrichment zone with a high concentration gradient at the protrusion tips, while the roots become zinc-depleted zones. According to the coupled mechanism of diffusion and electromigration, this non-uniform ion flux causes a localized surge in electric field intensity and reaction kinetics (Equation (2)) at the tips. This triggers a vicious cycle where “sharper tips grow faster,” a phenomenon known as the typical “tip growth effect ” [41]. 

Comparative analysis concludes that the HDES electrolyte, by modulating the coordination environment, achieves a homogeneous distribution of the Zn^2+^ concentration field. This effectively eliminates localized ion accumulation and the tip effect that typically trigger dendritic growth. Guided by a uniform ionic flux, the Zn anode surface undergoes smooth, layered growth. Even after prolonged deposition (300 s), only rounded protrusions are observed, successfully suppressing the formation of sharp dendrites.

**Figure 4:**
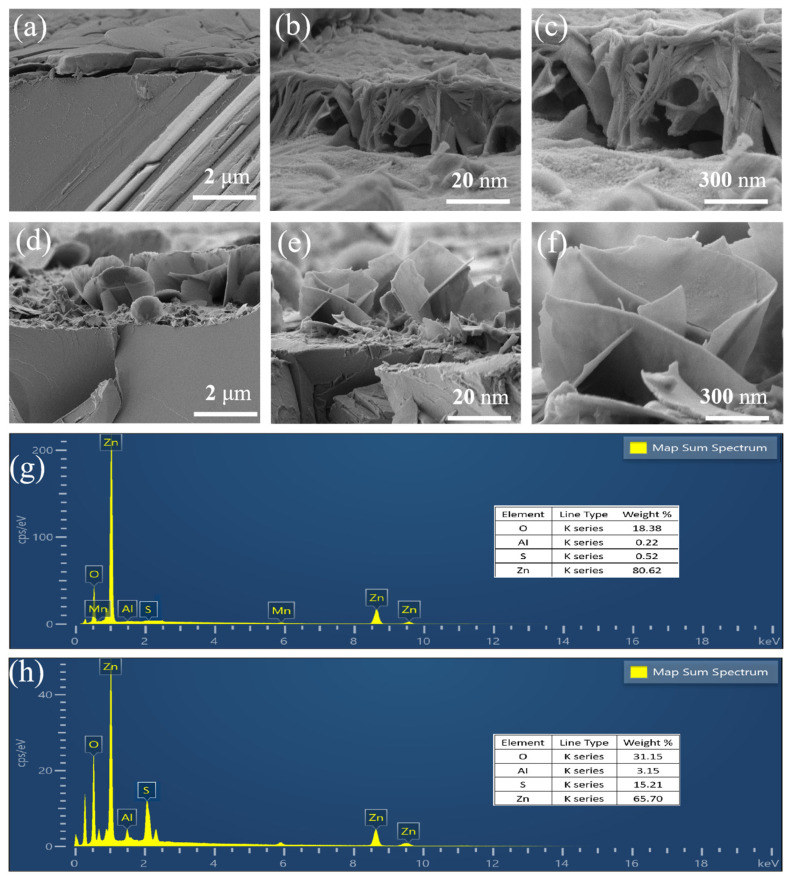
(**a**–**c**) SEM images of the Zn anode after reaction in the HDES system; (**d**–**f**) SEM images of the Zn anode after reaction in the aqueous ZnSO_4_ system; (**g**,**h**) EDAX images of the HDES system and the ZnSO_4_ system.

To validate the theoretical predictions derived from the phase-field simulations, ex situ SEM characterization was performed on the Zn anodes after cycling. As predicted by the simulation in Figure 3c, which indicated severe tip growth effects due to uneven ion flux, the experimental SEM images of the aqueous ZnSO_4_ system in Figure 4d–f exhibit a rough surface covered with large, sharp, and disordered hexagonal platelets. These sharp features correspond perfectly to the localized high-current-density regions identified in the concentration field simulation in Figure 3f. Figure 4d–f display the morphology of the Zn anode cycled in the ZnSO_4_ electrolyte system. The surface is covered by a thick, disordered layer of deposits, distinct from the smooth surface observed in the HDES system. Upon magnification, these deposits are identified as massive platelet-like crystals. These rigid platelets, characterized by extremely sharp edges, are stacked haphazardly with significant voids, and their dimensions are vastly larger than the nanosheets formed in the HDES. Under an electric field, these sharp edges induce a severe “tip effect,” attracting preferential Zn^2+^ deposition. This mechanism is identified as the primary culprit behind the rapid capacity decay and battery failure caused by dendrite-induced separator piercing.

In sharp contrast, and consistent with the smooth deposition predicted in Figure 2c, the Zn anode cycled in the HDES electrolyte displays a remarkably flat morphology, as shown in Figure 4a. The high-magnification images in Figure 4b,c reveal a unique interconnected 3D nanosheet network. This orderly and dense structure serves as direct experimental evidence that the HDES effectively homogenized the ion flux and eliminated the tip effect, thereby strictly validating the mechanism proposed by our phase-field models. Figure 4a–c present the post-cycling SEM images of the Zn anode in the HDES electrolyte system. At lower magnification, the anode surface exhibits a remarkably smooth and planar morphology, devoid of bulky protrusions, with the Zn deposition layer forming dense and intimate contact with the substrate. High-magnification views reveal a 3D porous network structure composed of interwoven nanosheets. This ordered arrangement of nanoscale sheets not only mitigates the risk of separator puncture but also significantly reduces the local effective current density due to its immense specific surface area. Consequently, this architecture alleviates electrode polarization and ensures superior stability at high rates. These findings demonstrate the exceptional capability of the DES electrolyte to modulate Zn^2+^ kinetics, facilitating abundant and uniformly distributed nucleation sites, thereby preventing localized ion accumulation.

To further probe the chemical composition of the deposition products, EDAX analysis was conducted, as shown in Figure 4g,h. For the pristine Zn anode, only characteristic Zn signals were detected. After cycling in the aqueous electrolyte, as shown in Figure 4h, the EDAX spectrum of the distinct hexagonal platelets showed intense signals corresponding to O and S elements. The atomic ratio suggests the formation of byproduct basic zinc sulfates, which aligns with the severe corrosion and passivation predicted by the theoretical analysis. Conversely, for the Zn anode cycled in the HDES system, shown in Figure 4g, the mapping results display a uniform and predominant distribution of Zn, with negligible O and S signals. This elemental evidence confirms that the unique nanosheet structure observed in SEM consists of high-purity metallic zinc, demonstrating the efficacy of the HDES in suppressing parasitic reactions and byproduct accumulation.

Collectively, these morphological results perfectly corroborate the simulation predictions, confirming that the HDES achieves a uniform zinc-ion flux by reconstructing the solvation sheath, thereby inducing this superior, dendrite-free nanosheet deposition morphology.

### 3.2. Structural Characterization and Physicochemical Properties

**Figure 5:**
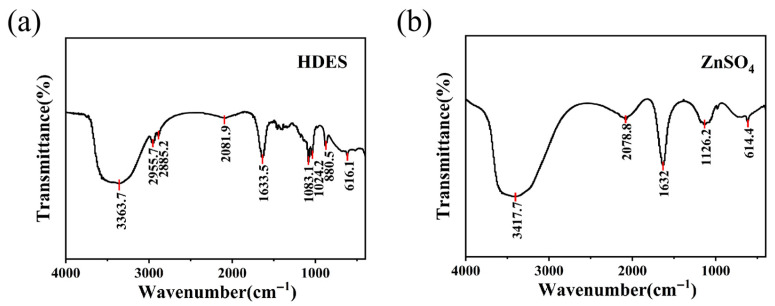
The FT-IR spectra of (**a**) the HDES and (**b**) the aqueous ZnSO_4_ solution.

Figure 5 illustrates the FT-IR spectra of the HDES and the aqueous ZnSO_4_ solution. As shown in Figure 5a, the hydroxyl (-OH) stretching vibration peak of the HDES exhibits a significant red shift of 54 cm^−1^, moving from 3417.7 cm^−1^ to 3363.7 cm^−1^. This shift indicates that the introduction of EG molecules facilitates the formation of a dense and robust hydrogen bonding network between EG, water molecules, and sulfate ions. Such strong intermolecular interactions weaken the force constant of the O-H bonds in water, leading to bond elongation and a subsequent decrease in vibrational frequency (lower wavenumber). Consequently, water molecules are effectively sequestered within the hydrogen bonding matrix by EG and the ions, which drastically reduces their thermodynamic activity, thereby suppressing the HER and electrode corrosion. New absorption bands emerging at 2955.7 cm^−1^ and 2885.2 cm^−1^ are attributed to the vibrational signals of the methylene (-CH_2_-) groups in the EG backbone, confirming the successful integration of the organic solvent as a primary electrolyte component. Furthermore, the bands in the 1000 cm^−1^–1200 cm^−1^ region become broadened and split, primarily at 1083.1 cm^−1^ and 1024.2 cm^−1^. This is mainly due to the superposition of the C-O stretching vibrations from EG and the shifted/deformed sulfate (SO_4_^2−^) signals [42,43]. This transformation suggests that (SO_4_^2−^) ions are no longer merely hydrated but are deeply incorporated into the Zn^2+^ solvation sheath, synergistically modulating the coordination environment of zinc ions alongside EG.

Figure 5b illustrates the typical FT-IR spectral features of hydrated zinc sulfate. The primary absorption bands at 1632 cm^−1^ and 3417 cm^−1^ originate from water of crystallization (lattice water). The presence of the O-H stretching vibration at such a high wavenumber indicates that water molecules in the aqueous solution exist predominantly in a free or unbound state. Meanwhile, the peaks within the 614–1126 cm^−1^ range are assigned to the characteristic vibrations of sulfate ions (SO_4_^2−^). The sharp peak profiles and the absence of significant splitting suggest that (SO_4_^2−^) maintains high molecular symmetry in the dilute solution. This indicates that the sulfate ions are primarily solvated by water molecules (forming hydrated sulfate) and exhibit relatively simple or weak interactions with the coordinating cations.

The distinct contrast between the two spectra clearly elucidates the molecular origin of the HDES electrolyte’s superior performance. By establishing a robust hydrogen bonding network to “sequester” free water and reconfiguring the ionic coordination environment, the HDES effectively mitigates parasitic side reactions triggered by the excessive water activity inherent in conventional aqueous electrolytes. These findings provide compelling microscopic evidence for achieving long-term cycling stability in zinc-ion batteries.

**Figure 6:**
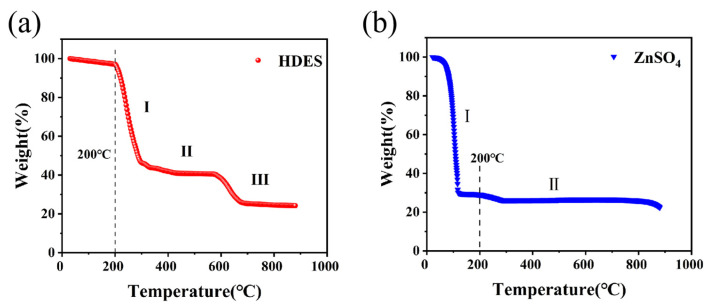
The thermogravimetric analysis results of (**a**) the HDES and (**b**) the aqueous ZnSO_4_ solution.

Figure 6a presents the TGA results, demonstrating that the as-prepared HDES exhibits negligible weight loss during Stage I (25–200 °C). This signifies the absence of free, volatile solvent water, confirming that the electrolyte effectively sequesters free water within a robust structural framework. Upon entering Stage II (200–600 °C), the weight undergoes a steady and gradual decline, eventually reaching approximately 20%. This broad weight loss region, spanning nearly 400 °C, corresponds to the progressive decomposition of various constituents (e.g., ethylene glycol, ZnSO_4_, and MnSO_4_) and their intermolecular interaction products. The significant broadening of the decomposition temperature window suggests that the components form a stable, integrated structure via synergistic interactions such as hydrogen bonding and coordination, requiring higher thermal energy for disruption. This indicates that the HDES electrolyte possesses exceptional thermal stability over a wide temperature range, outperforming most aqueous and aprotic electrolyte systems [44]. In Stage III (600–900 °C), the weight loss levels off and eventually stabilizes.

In contrast, the TGA curve of ZnSO_4_ in Figure 6b exhibits only two distinct stages. During Stage I (25–200 °C), the weight undergoes a sharp decline from 100% to approximately 40%, which is primarily attributed to the dehydration of lattice water (in the case of hydrated ZnSO_4_) and the initial thermal decomposition of ZnSO_4_ into zinc oxide (ZnO) and sulfur trioxide (SO_3_). In Stage II (200–900 °C), the weight decreases gradually before eventually reaching a stable plateau.

The aforementioned TGA results indicate that the prepared HDES exhibits no significant weight loss below 200 °C, corroborating the formation of a robust hydrogen-bonded network between the electrolyte components and water molecules, which effectively sequesters free water. Its broad and gradual decomposition temperature window reflects the strong intermolecular interactions and superior thermal stability of the system. This thermal robustness is highly consistent with the exceptional interfacial stability demonstrated by the electrolyte during electrochemical cycling.

**Figure 7:**
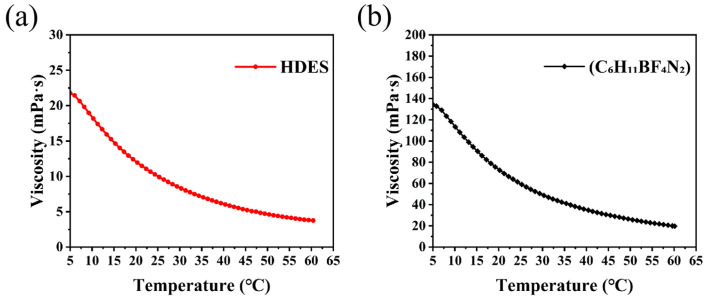
A viscosity analysis of (**a**) the HDES and (**b**) the ionic liquid.

Figure 7 illustrates the temperature dependence of viscosity for the HDES compared with the ionic liquid [(C_6_H_11_BF_4_)N_2_]. The deep eutectic electrolyte developed in this work exhibits significantly superior fluidity and wide-temperature adaptability. Within the range of 5 °C to 65 °C, the viscosity of the HDES remains consistently and substantially lower than that of the reference ionic liquid. Specifically, at 5 °C, the HDES possesses a viscosity of approximately 22 mPa·s, whereas the ionic liquid reaches a much higher value of 138 mPa·s. As the temperature rises to 65 °C, their viscosities steadily decline to approximately 4 mPa·s and 16 mPa·s, respectively.

This disparity originates from the structural characteristics of the systems: The HDES is composed of small molecules (e.g., ethylene glycol) organized through a dynamic hydrogen bonding network, resulting in weaker intermolecular forces and minimal steric hindrance. In contrast, the bulky and rigid organic cations in the ionic liquid lead to significantly higher flow resistance [45]. The low-viscosity characteristic endows the HDES electrolyte with accelerated ion migration and superior wettability toward both the electrodes and the separator, providing a crucial kinetic foundation for achieving low polarization and highly reversible electrochemical deposition behavior (as evidenced by the CV results).

Furthermore, the viscosity varies moderately with temperature, maintaining excellent fluidity even in the low-temperature region, which suggests great potential for applications across a broad temperature window, particularly in sub-zero environments. In summary, the viscosity analysis confirms the advantages of the HDES electrolyte in terms of ion transport and interfacial compatibility from a rheological perspective, providing a robust physical property basis for the enhancement of overall electrochemical performance. It is worth noting that the ionic conductivity of the viscous HDES electrolyte is generally lower than that of traditional dilute aqueous electrolytes. This is governed by the Stokes–Einstein relation, where the extensive hydrogen bond network within the HDES increases viscosity, thereby restricting the bulk mobility of Zn^2+^ ions and limiting the maximum achievable current density to some extent. However, this physicochemical property presents a necessary trade-off for stability. The reduced mobility and strong molecular interactions effectively suppress the thermodynamic activity of free water and inhibit the “tip effect” during deposition. Consequently, while the aqueous counterpart suffers from rapid failure due to dendrites and parasitic reactions despite its high conductivity, the HDES system sacrifices partial bulk transport speed to secure exceptional interfacial stability, enabling a dendrite-free morphology and a long lifespan exceeding 2000 cycles.

### 3.3. Electrochemical Performance

**Figure 8:**
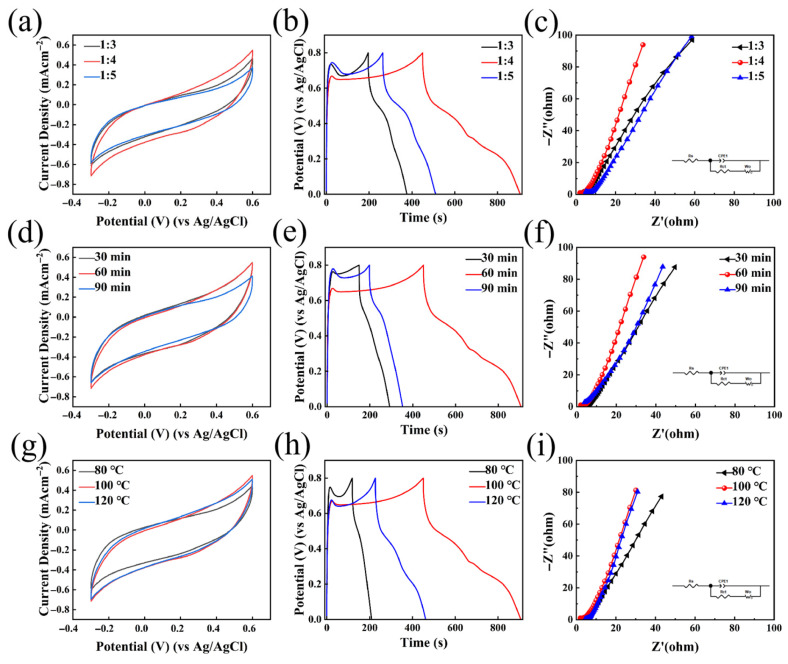
(**a**–**c**) The CV, GCD, and EIS curves of the HDES synthesized with various molar ratios (1:3, 1:4, and 1:5); (**d**–**f**) the CV, GCD, and EIS curves of the HDES synthesized for various durations (30, 60, and 90 min); (**g**–**i**) the CV, GCD, and EIS curves of the HDES synthesized at various temperatures (80, 100, and 120 °C).

Figure 8 illustrates the electrochemical performance of the HDES synthesized under various conditions using a three-electrode system. Figure 8a–c present the electrochemical results for samples with different molar ratios (1:3, 1:4, and 1:5). Figure 8a compares the CV curves at a scan rate of 10 mV s^−1^. It is observed that the 1:4 ratio yields the largest integrated area, indicating the highest specific capacitance. Figure 8b compares the GCD curves at a current density of 1 mA cm^−2^, where the 1:4 ratio exhibits the longest discharge duration. The EIS plots are shown in Figure 8c, with the equivalent circuit model provided in the inset. In this model, R_s_ and R_ct_ represent the series resistance and charge transfer resistance, respectively, associated with the double-layer capacitance and Faradaic resistance. The fitting parameters are listed in Table 1. The 1:4 ratio demonstrates the lowest impedance. The electrochemical performance of the HDES electrolyte is intrinsically governed by the molar ratio of the salt to the hydrogen bond donor (EG), which determines the delicate trade-off between physicochemical properties and the solvation environment. At the 1:3 ratio, the limited amount of EG is insufficient to fully dissociate the metal salts and disrupt the lattice energy, resulting in excessively high viscosity. This significantly hinders ion migration and mass transfer, leading to low ionic conductivity and large voltage hysteresis. Conversely, at the 1:5 ratio, the excess EG acts as a diluent. Although viscosity is reduced, the volumetric density of effective charge carriers (Zn^2+^) decreases, and the redundancy of solvent molecules may disrupt the optimal compactness of the hydrogen bond network, thereby compromising charge transport efficiency. The 1:4 ratio represents the optimal equilibrium point. At this specific stoichiometry, the system achieves the highest ionic conductivity by balancing carrier concentration and mobility. Furthermore, spectroscopic evidence suggests that the hydrogen bond network and Zn^2+^ solvation sheath are the most stable at this ratio, which minimizes the desolvation energy barrier and facilitates rapid interfacial kinetics, resulting in the maximum specific capacity and cycling stability observed.

Figure 8d–f display the electrochemical results for different synthesis durations (30, 60, and 90 min). As shown in the CV comparison (Figure 8d) at 10 mV s^−1^, the sample synthesized for 60 min possesses the maximum integrated area, reflecting the highest specific capacitance. Correspondingly, the GCD curves (Figure 8e) at 1 mA cm^−2^ show that the 60 min duration results in the longest discharge time. The EIS analysis (Figure 8f) confirms that the 60 min sample achieves the minimum impedance. The fitting parameters are listed in Table 1.

Figure 8g–i evaluate the influence of synthesis temperatures (80, 100, and 120 °C). At a scan rate of 10 mV s^−1^ (Figure 8g), the CV curve for the 100 °C sample shows the largest integrated area, signifying optimal capacitive performance. The GCD curves (Figure 8h) at 1 mA cm^−2^ further indicate that the 100 °C condition leads to the longest charging/discharging time. Consistent with these findings, the EIS results (Figure 8i) show that the sample synthesized at 100 °C exhibits the lowest R_s_ and R_ct_ values. The fitting parameters are listed in Table 1.

In summary, the comparative analysis demonstrates that the HDES synthesized with a 1:4 molar ratio at 100 °C for 60 min possesses the optimal electrochemical performance.

**Figure 9:**
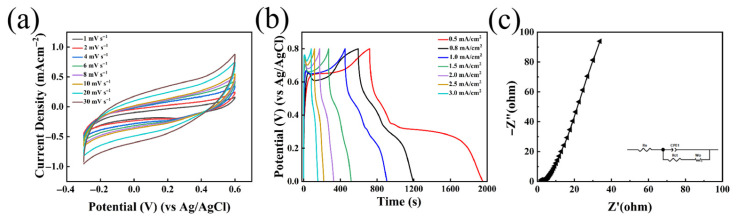
(**a**) The CV curves of the HDES at various scan rates; (**b**) the GCD curves of the HDES at current densities ranging from 0.5 to 3 mA cm^−2^; (**c**) EIS plots.

To further evaluate the electrochemical performance of the as-prepared HDES, electrochemical measurements were initially conducted using a standard three-electrode configuration. Figure 9a illustrates the CV curves of the HDES electrolyte at various scan rates ranging from 1 to 30 mV s^−1^. It is evident that all CV curves maintain a consistent shape without significant distortion as the scan rate increases, demonstrating excellent charge storage capability and highly efficient high-rate electrochemical response [46]. Figure 9b displays the GCD curves at current densities from 0.5 to 3 mA cm^−2^. All GCD profiles exhibit a highly symmetric charge/discharge process with discharge times decreasing in near-linear proportionality to the current density, indicating favorable electrochemical performance. The EIS results are presented in Figure 9c, with the equivalent circuit model provided in the inset. In this model, R_s_ and R_ct_ represent the series resistance and charge transfer resistance, respectively, which are associated with the double-layer capacitance and Faradaic resistance.

The viability of the HDES was further assessed by assembling asymmetric cells consisting of a Zn-Al LDH negative electrode and a MnO_2_ positive electrode. Parallel experiments were conducted by comparing the HDES-based system with a benchmark system employing a standard ZnSO_4_ electrolyte.

**Figure 10:**
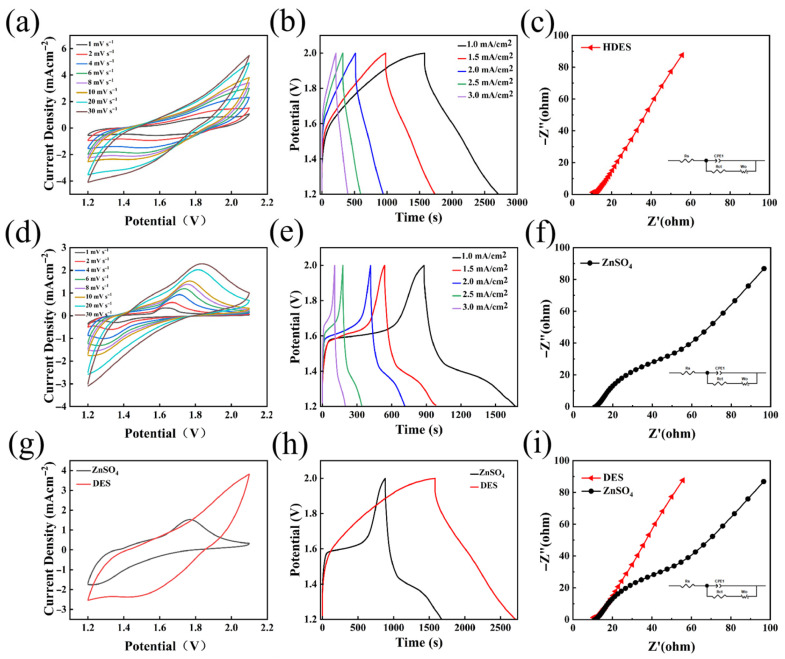
(**a**) The CV curves of the HDES-based device at various scan rates; (**b**) the GCD curves of the HDES-based device at current densities ranging from 0.5 to 3 mA cm^−2^; (**c**) the EIS plot of the HDES-based device; (**d**) the CV curves of the aqueous ZnSO_4_-based device at various scan rates; (**e**) the GCD curves of the aqueous ZnSO_4_-based device at current densities ranging from 1 to 3 mA cm^−2^; (**f**) the EIS plot of the aqueous ZnSO_4_-based device; (**g**) a comparison of CV curves for the HDES-based and aqueous ZnSO_4_-based devices at 10 mV s^−1^; (**h**) a comparison of GCD curves at 1 mA cm^−2^; (**i**) a comparison of EIS plots for the two devices.

Figure 10a illustrates the CV curves of the HDES-based device at various scan rates from 1 to 30 mV s^−1^. The CV profiles exhibit a well-defined quasi-rectangular or shuttle-like shape and maintain high morphological stability across all scan rates, indicating prominent pseudocapacitive characteristics. This suggests that the charge storage is primarily surface-controlled or near-surface-driven with minimal diffusion limitations. Even at a high scan rate of 30 mV s^−1^, the absence of significant distortion reflects rapid Zn^2+^ transport and reaction kinetics at the electrode interface, as well as low polarization, leading to superior rate capability. Furthermore, the smooth and highly symmetric redox profiles, devoid of sharp parasitic peaks, confirm the effective suppression of parasitic reactions such as the HER or byproduct formation [46]. Figure 10b displays the GCD curves within a current density range of 1 to 3 mA cm^−2^. All profiles exhibit regular triangular shapes with nearly identical charge and discharge slopes. This high degree of symmetry signifies excellent CE and electrochemical reversibility. The linear voltage–time relationship, characterized by the absence of distinct charge–discharge plateaus, is in excellent agreement with the CV results in Figure 10a, further confirming that charge storage is governed by rapid surface kinetics rather than being diffusion-limited. Moreover, the negligible voltage drop at the transition from charging to discharging indicates a low internal resistance. The EIS results for the HDES device are shown in Figure 10c. In the high-frequency region, the extremely small diameter of the semicircle reflects a minimal charge transfer resistance (R_ct_). The fitting parameters are listed in Table 2. The steep slope of the curve in the low-frequency region further indicates rapid Zn^2+^ migration at the electrode/electrolyte interface with minimal resistance. This is attributed to the optimized coordination environment mentioned earlier, which facilitates the desolvation of Zn^2+^ solvation shells for fast deposition. The inset provides the equivalent circuit model, where R_s_ and R_ct_ denote the series resistance and charge transfer resistance, respectively, while the constant phase element (CPE) and Faradaic resistance are also incorporated.

Figure 10d presents the CV curves for the device employing the aqueous ZnSO_4_ electrolyte across scan rates from 1 to 30 mV s^−1^. The profiles exhibit distinct redox peaks, signifying ion intercalation/deintercalation or phase transition reactions. Severe polarization is evident; as the scan rate increases from 1 to 30 mV s^−1^, the oxidation peak shifts toward higher potentials (right), while the reduction peak shifts toward lower potentials (left), leading to a significant expansion of the peak potential separation. This behavior is likely attributed to the accumulation of byproducts (e.g., basic zinc sulfate) on the electrode surface or the formation of a corrosion layer induced by free water, both of which obstruct ion transport. Consequently, the polarization becomes more pronounced at elevated current densities. Figure 10e displays the GCD curves within a current density range of 1 to 3 mA cm^−2^. The GCD profiles are non-linear and exhibit well-defined voltage plateaus for both charging and discharging processes. As the current density increases, the charging plateau shifts upward, and the discharging plateau shifts downward, resulting in substantial voltage hysteresis (overpotential). Notably, at 3 mA cm^−2^, the effective discharge time is drastically shortened, indicating that ion transport in the traditional aqueous electrolyte is significantly hindered, leading to severe polarization and rapid capacity fading at high rates. The EIS results for the ZnSO_4_-based device are shown in Figure 10f. A prominent semicircle with a large diameter is observed, signifying an extremely high charge transfer resistance. The fitting parameters are listed in Table 2. This suggests that the charge transfer kinetics at the electrode interface are highly sluggish with substantial impedance, indicating that the overall reaction rate is governed by diffusion-limited processes. The inset provides the equivalent circuit model, where R_s_ and R_ct_ represent the series resistance and charge transfer resistance, respectively, associated with the double-layer capacitance and Faradaic resistance, typically incorporating a CPE.

Figure 10g compares the CV curves of the HDES-based and aqueous ZnSO_4_-based devices at a scan rate of 10 mV s^−1^, providing definitive evidence of the HDES electrolyte’s superiority. Compared to the conventional ZnSO_4_ electrolyte, the HDES system exhibits a significantly larger integrated area, indicating a higher specific capacity. Furthermore, its characteristic quasi-rectangular profile reveals a transition in the charge storage mechanism toward surface-controlled pseudocapacitive behavior. This shift markedly enhances the reaction kinetics, effectively overcoming the issues of sluggish ion diffusion and severe polarization inherent in traditional aqueous electrolytes. Figure 10h shows the GCD comparison at a current density of 1 mA cm^−2^. The HDES system demonstrates a surface-controlled reaction characterized by a linear voltage–capacity relationship, which is highly conducive to high power output and prolonged cycle life. In contrast, the ZnSO_4_ system follows a diffusion-controlled process with distinct discharge plateaus, leading to substantial performance degradation at high rates. Under identical testing conditions (1 mA cm^−2^), the HDES-based device achieves a discharge duration of approximately 2700 s, significantly outperforming the aqueous system (1700 s). This enhanced capacity release is attributed to the optimized coordination environment of the HDES, which promotes the more efficient utilization of active sites. Figure 10i presents the EIS comparison, which directly proves that the HDES electrolyte significantly lowers the interfacial reaction energy barrier by restructuring the Zn-ion coordination environment. The minimal charge transfer resistance observed in the HDES system, as opposed to the massive R_ct_ in ZnSO_4_, indicates a substantial reduction in internal resistance. This ensures a minimal voltage drop and reduced thermal dissipation during high-current cycling, thereby elucidating the excellent triangular GCD symmetry and superior rate capability previously shown in Figure 10b.

It is noteworthy that the electrochemical profiles of the Zn–MnO_2_ cells differ significantly between the two electrolytes, indicating a fundamental shift in the charge storage mechanism. In the aqueous ZnSO_4_ electrolyte, the CV curves display sharp redox peaks, and the GCD profiles show distinct voltage plateaus. These features correspond to a diffusion-controlled battery-type mechanism, dominated by the bulk intercalation/extraction of Zn^2+^/H^+^ ions and accompanied by phase transformations (e.g., the reversible formation of basic zinc sulfates). The significant peak separation observed reflects high polarization and sluggish kinetics due to the accumulation of resistive byproducts on the electrode surface. Conversely, in the HDES electrolyte, the CV curves exhibit a quasi-rectangular shape without sharp peaks, and the GCD profiles present a linear, triangular slope. These characteristics are indicative of surface-controlled pseudocapacitive behavior. The HDES electrolyte, with its unique solvation structure and reduced water activity, effectively suppresses the formation of insulating passivation layers, thereby maximizing the exposure of surface active sites. Consequently, the dominant storage mechanism transitions from slow bulk diffusion to fast surface Faradaic reactions. This pseudocapacitive mechanism minimizes structural strain during cycling and facilitates rapid ion transport, aligning with the observed superior rate capability and ultralong cycling stability.

**Figure 11:**
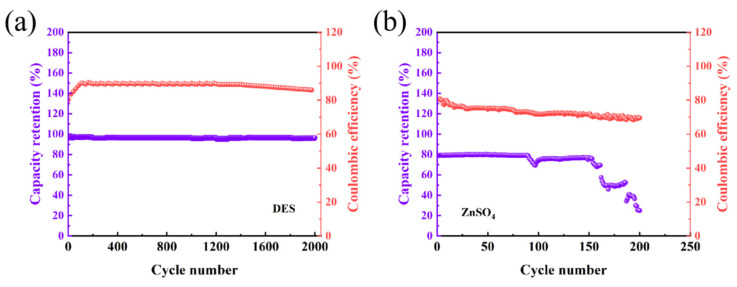
Capacity retention and Coulombic efficiency curves at a current density of 3 mA cm^−2^ for (**a**) the HDES-based device and (**b**) the aqueous ZnSO_4_-based device.

Figure 11a presents the capacity retention and CE curves of the HDES-based device at a current density of 3 mA cm^−2^. Throughout the 2000-cycle test, the capacity retention consistently remains above 95%, indicating negligible capacity fading. This demonstrates the structural integrity of the electrode material and the homogeneous deposition of zinc. Following an initial activation phase, the CE rapidly stabilizes at a high level with minimal fluctuations, reflecting superior electrochemical reversibility. This confirms that the HDES electrolyte effectively suppresses parasitic side reactions, particularly the HER.

In contrast, Figure 11b displays the corresponding results for the aqueous ZnSO_4_ device. A gradual decline in capacity retention is observed within the first 90 cycles, followed by a sharp capacity plunge between the 90th and 100th cycles, typically attributed to dendrite-induced micro-short circuits or the accumulation of insulating byproducts. By the 150th cycle, the capacity collapses to below 40% with severe oscillations, signaling total cell failure. Furthermore, the CE shows a continuous downward trend and intense fluctuations after 150 cycles, indicating the sustained consumption of the electrolyte and active zinc through irreversible side reactions.

It is noted that the CE of the full cells presents a distinct difference between the two systems. In the aqueous ZnSO_4_ electrolyte, the CE fluctuates at a low level (<80%), which is primarily attributed to the severe dissolution of the MnO_2_ cathode (loss of active mass) and the HER on the Zn anode. The continuous consumption of electrolyte and active species leads to the observed rapid capacity decay. In contrast, the HDES electrolyte enables a significantly higher and more stable CE (90%). While the CE is not yet 100% due to the intrinsic irreversibility of Mn-based intercalation chemistry and minor side reactions, the substantial improvement over the aqueous system serves as robust evidence for the electrolyte’s efficacy. The HDES constructs a water-deficient environment that effectively suppresses Mn dissolution and the HER, thereby securing the integrity of the interface and supporting the ultralong lifespan of over 2000 cycles. The cycling performance and capacity retention of other zinc-ion batteries are compared and presented in Table 3.

The comparison of Figure 11a,b validates that the EG in the HDES forms a complex deep eutectic coordination structure with ZnSO_4_ and MnSO_4_, where water molecules are strongly sequestered within a robust hydrogen bonding network. The restricted water activity effectively eliminates the possibility of the HER. Without the coverage of passivating byproducts, the electrode surface remains electrochemically active, thereby preserving a zincophilic interface. Moreover, the reconfigured solvation shell in the HDES promotes a transition from disordered to ordered Zn^2+^ deposition, fundamentally mitigating the risk of dendrite growth and subsequent short circuits.

## 4. Conclusions

In summary, this study presents a high-performance HDES electrolyte system based on ZnSO_4_, MnSO_4_, and EG to overcome the intrinsic instabilities of AZIBs. By reconfiguring the Zn^2+^ solvation environment, the HDES effectively homogenizes the ionic flux and electric field distribution, thereby eliminating the “tip effect” and facilitating a transition from disordered dendritic growth to a dense, ordered 3D nanosheet architecture. This mechanism, supported by phase-field simulations and SEM characterization, ensures high reversibility and stability, achieving a capacity retention of over 95% across 2000 cycles. Future research should focus on balancing ionic conductivity and viscosity through molecular engineering while expanding compatibility with high-voltage cathodes to maximize energy density. Additionally, employing advanced in situ characterization (e.g., cryo-EM and NMR) will be crucial to unraveling molecular-level interfacial dynamics. Broadly, this solvation-tuning strategy offers a scalable roadmap for other multivalent systems (Mg, Al-ion batteries). We anticipate that task-specific HDES electrolytes will drive the development of high-safety, long-life energy storage devices for large-scale grid and extreme-environment applications.

## Figures and Tables

**Table 1:** EIS fitting parameters of EIS curves for HDES synthesized under different molar ratios (1:3, 1:4, and 1:5), reaction times (30, 60, and 90 min), and temperatures (80, 100, and 120 °C).

	1:3	1:4	1:5	30 min	60 min	90 min	80 °C	100 °C	120 °C
R_s_	3.23	2.01	4.97	3.67	2.01	3.87	3.05	2.01	4.33
R_ct_	1.21	0.68	4.32	3.10	0.68	1.25	2.05	0.68	2.45

**Table 2:** EIS fitting parameters of EIS curves for HDES and ZnSO_4_ systems.

	HDES	ZnSO_4_
R_s_	10.32	11.19
R_ct_	0.89	26.81

**Table 3:** Performance comparison table of zinc-ion batteries.

System	Number of Cycles	Capacitance Retention (%)	Ref.
SITL	2000	86%	[47]
MXene	500	81%	[48]
MCE	500	90.67%	[49]
FCDs	2000	81.4%	[50]
DES	2000	95%	This work

## Data Availability

The data presented in this study are available on request from the corresponding author.
